# Examining the Pathogenesis of MAFLD and the Medicinal Properties of Natural Products from a Metabolic Perspective

**DOI:** 10.3390/metabo14040218

**Published:** 2024-04-12

**Authors:** Yansong Fu, Zhipeng Wang, Hong Qin

**Affiliations:** Department of Nutrition and Food Hygiene, Xiangya School of Public Health, Central South University, Changsha 410006, China; 226901016@csu.edu.cn (Y.F.); 226911033@csu.edu.cn (Z.W.)

**Keywords:** metabolic-associated fatty liver disease, nutrients, metabolism, natural products

## Abstract

Metabolic-associated fatty liver disease (MAFLD), characterized primarily by hepatic steatosis, has become the most prevalent liver disease worldwide, affecting approximately two-fifths of the global population. The pathogenesis of MAFLD is extremely complex, and to date, there are no approved therapeutic drugs for clinical use. Considerable evidence indicates that various metabolic disorders play a pivotal role in the progression of MAFLD, including lipids, carbohydrates, amino acids, and micronutrients. In recent years, the medicinal properties of natural products have attracted widespread attention, and numerous studies have reported their efficacy in ameliorating metabolic disorders and subsequently alleviating MAFLD. This review aims to summarize the metabolic-associated pathological mechanisms of MAFLD, as well as the natural products that regulate metabolic pathways to alleviate MAFLD.

## 1. Introduction

Non-alcoholic fatty liver disease (NAFLD) represents a spectrum of disorders caused by excessive accumulation of fat in the liver (defined as hepatic steatosis ≥ 5%) in the absence of alcoholic over-consumption or other chronic liver diseases, ranging from simple steatosis to steatohepatitis, fibrosis, cirrhosis, and hepatocellular carcinoma [1,2]. NAFLD is considered to be a metabolic disease caused by a complex interaction among genetic factors, systematic metabolic disorders, and the environment, and factors such as race, genetic susceptibility, dietary habits, metabolic conditions, immunity, and intestinal flora are closely related to its clinical performance and disease progression [3]. Recently, the term metabolic-associated fatty liver disease (MAFLD) has been considered to be more in line with the characterization of the syndrome and represents more significant clinical, research and patient benefits [4,5]. With an estimated prevalence of 39.22% globally, MAFLD has been the most common liver disease in the world [6,7].

The liver functions as a crucial metabolic organ in the body and metabolic disorders in the liver play a key role in the progression of various hepatic diseases. It is responsible for synthesizing, metabolizing, storing, and redistributing carbohydrates, proteins, and lipids, in which glucose and lipid metabolism are of paramount importance in the pathophysiology of MAFLD [8]. Metabolic disorders, including insulin resistance, impaired glycemic control, altered lipid metabolism, and changes in amino acid composition, are considered the main pathogenesis of MAFLD [9]. The pathogenic role of macronutrient metabolic imbalances in MAFLD has been well established. Additionally, micronutrients, namely, vitamins and minerals, also play a significant role, as the liver is also highly responsible for the metabolism of micronutrients. Specifically, metabolism imbalances of vitamins (vitamins A, D, E, etc.) and minerals (iron, copper, zinc, etc.) have been linked to the development of MAFLD [10]. Therefore, MAFLD involves the dysregulation of multiple nutrient metabolic pathways and significant etiologic heterogeneity, which poses a challenge in exploring and understanding the pathogenesis of MAFLD.

Currently, there are no drugs approved for clinical use in MAFLD. It is shown that an abnormal lifestyle has a strong connection with the disease’s progression. Recent evidence further emphasized that a shift in dietary patterns, especially toward a high-fat and/or a high-sugar diet, highly contributes to the onset of MAFLD by interfering with the metabolic functions and homeostasis of the body [11,12]. However, maintaining an improved lifestyle including eating habits is often challenging for patients with MAFLD, which causes weakened clinical benefits. Hence, it is crucial to investigate the pathogenesis of MAFLD thoroughly and identify safe and effective prevention and treatment strategies. In recent years, natural active substances, mainly of plant origin, have attracted extensive attention and have become an important source for developing therapies for various diseases [13]. Numerous studies have reported promising applications of natural products in preventing and controlling MAFLD. Therefore, this review aims to summarize the association between nutrient metabolism disorders and MAFLD, as well as the natural products that can potentially prevent and treat MAFLD by modulating nutrient metabolism disorders.

## 2. Nutrient Metabolism and MAFLD

### 2.1. Macronutrient Metabolism and MAFLD

#### 2.1.1. Lipid Metabolism and MAFLD

The liver plays a crucial role in regulating lipid metabolism via complex molecular mechanisms. It is highly involved in lipid digestion, absorption, uptake, synthesis, secretion, and oxidation. Hepatic steatosis occurs when the pathways of lipid uptake and synthesis are stronger than the pathways of lipid oxidation and export [14]. Lipid metabolism disorder is the most critical pathogenesis of MAFLD.

##### Lipid Uptake and MAFLD

**Figure 1:**
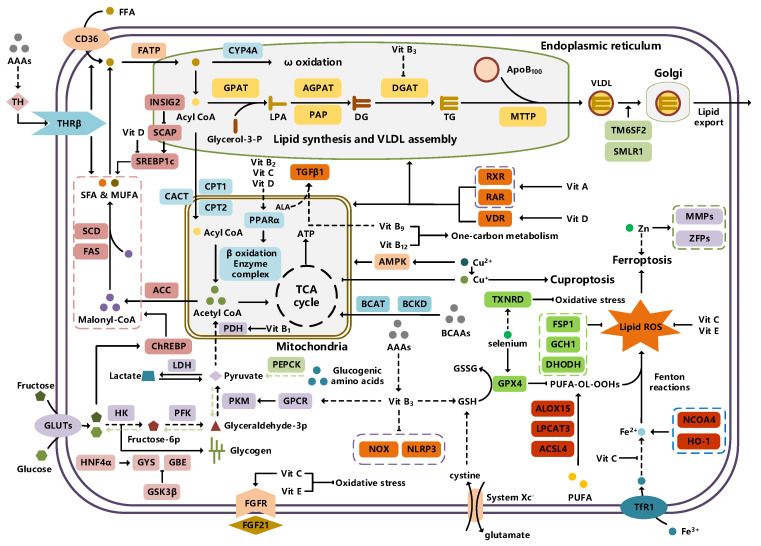
An illustration of the nutrient metabolism pathways involved in MAFLD.

Typically, adipocytes store lipids from dietary sources to maintain normal blood lipid levels. However, in pathological conditions such as obesity and metabolic syndrome that lead to insulin resistance, lipolysis in adipocytes is increased, resulting in the release of large amounts of free fatty acids into the bloodstream [15]. Hepatocytes subsequently increase fatty acid uptake in response, which is mainly mediated by lipid transporters such as Cluster Determinant 36 (CD36) and Fatty Acid Transporter Proteins (FATPs) (Figure 1). CD36 is mainly located at the plasma membrane and plays important roles in hepatic lipid transport, oxidation, and synthesis, which are regulated by various upstream factors such as Peroxisome Proliferator-Activated Receptor γ (PPARγ) and Arylhydrocarbon Receptor (AhR) [15,16]. After palmitoylation, CD36 is able to recognize and capture fatty acids, followed by depalmitoylation to initiate endocytosis to transport fatty acids into cells [17]. CD36 localization to the plasma membrane increases in the steatohepatitis phase, and the inhibition of CD36 palmitoylation promotes its translocation to the mitochondria, thereby ameliorating hepatic lipid metabolism disorders in mice and reducing inflammatory responses [18,19]. Therefore, CD36 could be a crucial and promising therapeutic target for MAFLD. FATPs contain six members of FATP1-6 encoded by *SLC27A1-6*, respectively, which are essential for the transport of long-chain fatty acids [20]. FATP2/5 are the major isoforms, of which FATP5 is unique in liver [20,21]. Evidence suggested that upregulated FATP2 promoted hepatic fat accumulation and could be one of the potential therapeutic targets for hepatic steatosis [22,23]. In addition, upregulated FATP5 was associated with increased hepatic steatosis in male patients with MAFLD [24]. FATP5 knockdown effectively alleviated obesity and hepatic steatosis in mice [25,26]. Notably, downregulated FATP5 may be a risk factor for advanced MAFLD [27,28]. However, the dynamic regulatory mechanisms of fatty acid transporters in the liver remain unclear, and further investigation is needed to identify their roles in different MAFLD stages and develop the corresponding targeted strategies.

##### De Novo Lipogenesis and MAFLD

De novo lipogenesis (DNL) is a complex metabolic pathway that converts excess carbohydrates into fatty acids [29]. The liver is an important organ for carrying out DNL. Briefly, acetyl-CoA derived from carbohydrates is converted to long-chain saturated fatty acids by Acetyl-CoA Carboxylase (ACC) and Fatty Acid Synthase (FAS), which can be subsequently converted to mono-unsaturated fatty acids by Stearoyl-CoA Desaturase 1 (SCD1) [30]. The neo-synthesized fatty acids are stored in the liver as triglycerides or doped into VLDL for extrahepatic export [30]. The DNL pathway is essential for maintaining energy homeostasis and glucose–lipid metabolic homeostasis (Figure 1). However, over-activated hepatic DNL pathways are important in MAFLD pathogenesis [29,30]. Carbohydrate Response Element Binding Protein (ChREBP) is one of the key transcription factors of hepatic DNL, which drives the transcriptional expression of crucial DNL enzymes such as ACC, FAS, and SCD [31,32]. Numerous studies have shown that a high-sugar diet can induce MAFLD. In particular, a high fructose diet can induce more severe steatohepatitis [33]. This is largely due to the activation of the ChREBP pathway, which in turn enhances the hepatic DNL pathway [34,35]. A recent study revealed the positive role of the activated NF-κB p65/Sorcin signaling pathway in ChREBP-mediated hepatic DNL and thus MAFLD induced by a high-sugar diet [36]. The degradation of key regulators of DNL such as ChREBP via ubiquitination can inhibit hepatic steatosis [37]. However, it is noteworthy that ChREBP knockout mice fed a high-sugar diet exhibited fructose intolerance, malabsorption, and gastrointestinal symptoms, which may be related to the homeostasis imbalance of the intestinal microenvironment [38,39]. Overall, ChREBP is an important target against MAFLD. Given its crucial physiological function, it is necessary to further consider and weigh the pros and cons of intervention therapies targeting ChREBP.

Another key transcription factor of the DNL pathway is Sterol Regulatory Element Binding Proteins (SREBPs). SREBPs consist of SREBP1a, SREBP1c, and SREBP2, with SREBP1c playing a key role in the trophic regulation of fatty acids and triglycerides in lipid-forming organs such as the liver [40]. SREBP1 is positively regulated by insulin, Liver X Receptors (LXRs), and other signaling pathways and is subsequently cleaved by SREBP Cleavage-Activating Protein (SCAP) in the endoplasmic reticulum membrane and translocated to the Golgi apparatus for activation [41]. Activated SREBP1c then enters the nucleus and regulates the transcription of DNL-related genes, including ACC and FAS [42]. Therefore, inhibition of the SCAP/SREBP1 pathway is one of the potential therapeutic strategies for MAFLD (Figure 1). Recent studies have found that Flavin-containing Monooxygenase 2 (FMO2) competitively binds SCAP, thereby inhibiting SREBP1 activation and ameliorating steatosis, inflammation, and fibrosis [43]. The unique role of CD36 in regulating DNL has also been recently reported. CD36 activation by insulin disrupts the binding of SCAP to Insulin-Induced Gene 2 (INSIG2), which activates the SREBP1 pathway [44]. In addition, studies have also revealed that Caspase-2 activates the SREBP1/2 pathway instead of the SCAP-dependent mechanism, thereby exacerbating MAFLD lesions [45,46]. However, it should not be overlooked that the liver-specific knockdown of SCAP and thus inhibition of SREBP exacerbated liver injury, fibrosis, and carcinoma in steatohepatitis mice instead, which may be caused by the disruption of phospholipid metabolic homeostasis [47]. This provides a new perspective for understanding the pathophysiology of SREBP. In conclusion, the DNL pathway is an indispensable physiological function, and interventions targeting the key regulators of the hepatic DNL pathway need to be further evaluated for their necessity and safety, which will be significant for avoiding their risks and side effects.

##### Fatty Acid Oxidation and MAFLD

Fatty acids are important energy substances and can release a large amount of energy stored in ATP via enzyme-controlled oxidation reactions. Mitochondrial β-oxidation is the main form of fatty acid oxidation (FAO) [48]. Short-, medium-, and long-chain fatty acids are converted to lipoacyl-CoA and then enter the mitochondria with the help of Carnitine Palmitoyl Transferase 1 (CPT1), Carnitine Acyl Carnitine Translocase (CACT), and Carnitine Palmitoyl Transferase 2 (CPT2) successively to generate abundant acetyl-CoA through a series of enzymatic reactions (Figure 1) [48,49]. Ultra-long-chain fatty acids, dihydroxy and trihydroxy cholesteric acids, long-chain dicarboxylic acids, and certain poly-unsaturated fatty acids need to be pre-processed in peroxisome before being translocated into the mitochondria to complete thorough β-oxidation [50]. In addition, long-chain and ultra-long-chain fatty acids can be metabolized to dicarboxylic acids by cytochrome P450 (CYP4A)-catalyzed ω-oxidation in the endoplasmic reticulum to provide substrates for peroxisomal β-oxidation (Figure 1) [51]. Peroxisome proliferator-activated receptor α (PPARα) plays a key role in the regulation of the FAO systems above [52]. The blockage of FAO, which results in the high intracellular accumulation of fatty acids, is an important pathogenic mechanism of MAFLD. The impaired mitochondrial function of FAO in the liver is observed in patients with MAFLD and is positively associated with the syndrome [53]. Therefore, improving impaired hepatic FAO is significant for the treatment of MAFLD.

A number of studies have characterized and identified targets associated with abnormal hepatic FAO function in MAFLD. The hepatic acetylation level of Acyl-CoA Synthetase Long-chain family member 5 (ACSL5) and expression levels of Acyl-CoA Synthetase Long-chain family member 4 (ACSL4) were increased in patients and mouse models with MAFLD, and the inhibition of ACSL5 acetylation and ACSL4 expression enhanced hepatic mitochondrial FAO and reduced lipid accumulation [54,55]. In addition, hepatic Methylation-Controlled J protein (MCJ) levels were elevated in patients with MAFLD, and the inhibition of MCJ enhanced mitochondrial FAO and alleviated liver injury and fibrosis [56]. A recent study also reported that hepatic Eukaryotic Initiation Factor 5A (EIF5A) was downregulated in patients and mice with MAFLD, and restoration of EIF5A levels ameliorated impaired mitochondrial FAO and blocked MAFLD progression [57]. Interestingly, CD36 also plays an important role in regulating FAO. The palmitoylation level of CD36 was upregulated in MAFLD, and the inhibition of palmitoylation increased the distribution of CD36 across the mitochondrial membrane, which enhanced mitochondrial FAO and alleviated MAFLD [58]. Overall, hepatic FAO is dysfunctional in MAFLD, and the restoration of impaired FAO is one of the potential therapeutic strategies for MAFLD.

##### Lipid Export and MAFLD

Another important function of the liver for regulating lipid metabolism is synthesizing and secreting triglyceride (TG)-rich Very Low-Density Lipoprotein (VLDL), which is essential for maintaining hepatic lipid homeostasis. The first step in hepatic lipid export is the synthesis of triglycerides. After conversion to lipoyl-CoA, exogenous or neo-synthesized fatty acids can be used as raw materials to synthesize TG. Glycerol-3-Phosphate Acyl Transferase (GPAT) catalyzes the formation of lysophosphatidic acid (LPA) from lipoyl-CoA and glycerol-3-phosphate (G3P), which subsequently synthesize diethylene glycol (DG) by Acylglycerol-3-Phosphate Acyl Transferases (AGPATs), Phosphatidic Acid Phosphatases (PAPs) and ultimately synthesize TG through diacylglycerol acyltransferase (DGAT) [59]. TGs further serve as the core lipid component of VLDL (Figure 1) [60]. DGAT may serve as a promising therapeutic target for MAFLD [61]. The knockdown of DGAT2 significantly reduced diet-induced hepatic TG content and improved hepatic steatosis in mice [62]. Therefore, blocking hepatic TG synthesis may contribute to alleviating MAFLD, but its feasibility and safety remain to be further evaluated.

Disturbances in hepatic VLDL assembly and secretion lead to excessive accumulation of TG and cholesterol, which drive the progression of MAFLD [63,64]. Microsomal Triglyceride Transfer Protein (MTTP) in the endoplasmic reticulum catalyzes the lipidation (i.e., incorporation of TG) of Apolipoprotein B_100_ (ApoB_100_), the precursor of VLDL, which is subsequently translocated to the Golgi apparatus for maturation (Figure 1) [63]. Therefore, promoting VLDL assembly and secretion represents a potential therapeutic strategy for MAFLD. Transmembrane 6 Superfamily member 2 (TM6SF2) is localized at the smooth endoplasmic reticulum and promotes VLDL secretion by assisting in ApoB lipidation [65,66]. Another recent study has identified the role of Small Leucine-Rich protein 1 (SMLR1), which is specifically expressed in the liver, in the regulation of VLDL secretion, with SMLR1 deficiency leading to hepatic lipid accumulation [67]. In addition, intracellular phospholipid metabolism has an important impact on hepatic VLDL output [68]. Recent studies have further shown that reduced hepatic phosphatidylcholine (PC) and phosphatidylethanolamine (PE) levels are important features of lipid metabolism imbalance in MAFLD, and the restoration of PC and PE levels can effectively improve impaired hepatic VLDL secretion and alleviate lipid accumulation [69,70,71]. Recent studies have also reported that mammalian Target of Rapamycin Complex 1 (mTORC1) enhanced VLDL output via the activation of Cytidine triphosphate: phosphocholine Cytidylyltransferase-α (CCTα) and alleviated steatohepatitis in mice [72]. Notably, extrahepatic tissues also play an important role in regulating hepatic lipid output. Leptin secreted by adipose tissue can stimulate hepatic VLDL output via the brain–vagus–liver axis and alleviate hepatic steatosis [73,74]. In contrast, leptin levels are reduced in patients with MAFLD compared with healthy populations [75]. This further suggests that MAFLD is a multi-systemic metabolic disease and also provides a novel idea for the treatment of MAFLD. However, over-activation of hepatic lipid output may result in other adverse consequences, including atherosclerosis due to elevated plasma apolipoproteins [76]. This poses a new challenge for the development of interventional approaches targeting hepatic lipid export pathways.

##### Lipidomic Profiling, Lipotoxicity, and MAFLD

Lipidomics studies lipid molecular networks and metabolic pathways at the cellular, tissue, and biosystem levels [77]. Differences in serum lipidomic profiles at different MAFLD stages have provided new perspectives and evidence for the assessment and classification of the syndrome [78]. Lipidomic analysis showed that serum free fatty acids, TG, ceramides, and bile acid levels were significantly elevated in patients with MAFLD, and hepatic saturated fatty acids and poly-unsaturated fatty acid levels were also significantly higher than those in healthy people [79]. Serum acylcarnitines, sphingolipids, monoglycerides, linoleic acid, and some phosphatidylcholine levels were decreased, while diglycerides, other phosphatidylcholines, phosphatidylinositol, and phosphatidylethanolamine levels were increased in female patients with obesity and MAFLD [80]. In another study, the relative levels of serum diglycerides, TG, phosphatidylinositol, and dihydroceramides were significantly increased in patients with MAFLD, whereas the levels of phosphatidylglycerol, phosphatidylcholine, phosphatidylserine, lysophosphatidylcholine, and cholesteryl esters were decreased [81]. The fact that the progression of MAFLD is accompanied by a tendentious change in serum lipidomic profiles has provided convenience for its noninvasive and accurate diagnosis. Given the metabolic heterogeneity, further studies are needed to determine the applicability of serum lipid metabolites as the marker at different pathological stages.

Changes in serum lipidomic profiles reflect, in one respect, the central role of lipids in MAFLD progression, and understanding the impact of hepatic lipids contributes to clarifying the pathogenesis of MAFLD. In general, TGs accumulated in the liver during the steatosis stage of MAFLD represent an inert storage form of toxic lipids, while large amounts of free fatty acids, cholesterol, and their metabolites induce lipotoxicity [82]. Toxic lipids are closely associated with MAFLD progression by disrupting organelle structure and function, leading to cellular damage and even cell death [83]. The endoplasmic reticulum, as a membrane organelle rich in lipid components, is significantly affected by lipotoxicity. Large amounts of toxic lipids initiate the unfolded protein response pathway through endoplasmic reticulum stress, which in turn leads to MAFLD via upregulating the DNL pathway, activating inflammasomes, and inducing hepatocyte apoptosis [84,85,86]. The inhibition of over-activated endoplasmic reticulum stress in hepatocytes represents a novel therapeutic strategy for MAFLD [87]. As mentioned above, mitochondria are the primary place for β-oxidation of fatty acids, which is also an important target organelle of lipotoxicity. In MAFLD, sustained fatty acid and acetyl-CoA fluxes beyond the processing limits of mitochondria in the liver lead to the uncontrolled generation of reactive oxygen species (ROS), which in turn leads to the reprogramming of hepatic lipid metabolism, insulin resistance, and inflammation [88,89]. Hence, improving impaired mitochondrial structure and function may contribute to alleviating MAFLD.

#### 2.1.2. Carbohydrate Metabolism and MAFLD

The liver also plays a central role in carbohydrate metabolism via the uptake and storage of elevated blood glucose after feeding and the production and release of glucose during fasting. Carbohydrate metabolism in the liver is regulated by a variety of physiological mechanisms, such as hormone (insulin, glucagon, etc.) homeostasis, allosteric control by metabolites (acetyl coenzyme A, glucose, glucose-6-phosphate, etc.), substrate availability, and cellular redox status [90]. Disturbed hepatic carbohydrate metabolism is another important pathogenesis of MAFLD.

##### Carbohydrate Uptake and MAFLD

Carbohydrates such as glucose and fructose function as an important source of energy for the majority of cells in the body and important substrates for metabolic pathways such as de novo lipogenesis. Cells take up exogenous carbohydrates mainly through Glucose Transporter proteins (GLUTs/SLC2As), of which GLUT2, GLUT5, GLUT8, etc., are highly expressed in hepatocytes (Figure 1) [91]. GLUT2 is one of the key components for uptaking extracellular glucose. Protease-Activated Receptor 2 (PAR2) inhibits the FoxA3-dependent expression of GLUT2, leading to impaired hepatic glucose uptake and insulin resistance, which drives MAFLD progression [92]. This suggests the complexity of hepatic metabolism and MAFLD pathogenesis. In addition, a recent study reported that Sodium-dependent Glucose Transporter protein 2 (SGLT2) was significantly upregulated in steatohepatitis, suggesting that there is a metabolic adaptive shift in glucose transporters, and the inhibition of SGLT2 attenuated lipid accumulation, inflammation, and fibrosis, which may be related to reduced O-GlcNAcylation and increased autophagic flux in hepatocytes [93]. Similarly, SGLT2 inhibitors administered in combination with PPARα regulators prevented hepatocellular carcinogenesis in advanced MAFLD [94]. As mentioned above, high fructose intake can lead to MAFLD. GLUT8 is a key component in fructose uptake in hepatocytes and plays a crucial role in fructose-induced steatosis [95]. A recent study reported that Transmembrane 4L Six Family member 5 (TM4SF5) regulated the localization and activation of GLUT8 through transient binding, resulting in fructose-driven hepatocellular lipogenesis [96]. In contrast, upregulated TM4SF5 mediates the inflammatory response by promoting glucose uptake, glycolysis, and glucose sensitivity leading to hepatic macrophage M1 activation in MAFLD [97]. In conclusion, further studies are necessary to determine the pathological roles and metabolic shifts in various glucose transporters in MAFLD progression, which is of great significance in understanding the pathogenesis of MAFLD.

##### Glycogen Metabolism and MAFLD

The liver is one of the major places for glycogen synthesis and storage. Glycogen metabolism functions as an important physiological regulatory mechanism for maintaining carbohydrate homeostasis. Hyperglycemia damages hepatocytes and pancreatic β-cells, leading to hepatic insulin resistance, which induces and exacerbates MAFLD [98]. Glucose is transformed into glycogen through the processes of phosphorylation, metastasis, pyrophosphorylation, polymerization, and branching sequentially catalyzed by glucokinase (GCK), glycogen synthase (GYS), and glycogen branching enzyme (GBE), while synthesized glycogen is dissociated into glucose for utilization catalyzed by enzymes, such as glycogen phosphorylase, glycogen debranching enzyme, and glucose-6-phosphate translocase (Figure 1) [99]. Actually, disturbances in glycogen metabolism are common in the hepatocytes of patients with MAFLD [100]. The decreased synthesis or enhanced catabolism of glycogen indicates an increased glucose flux to other pathways including de novo lipogenesis, thereby exacerbating hepatic steatosis. A number of studies have identified upstream targets associated with disturbed glycogen metabolism in MAFLD. Bone morphogenetic protein 4 (BMP4) can inhibit adipogenesis by activating the mTORC2 pathway to promote the expression of glycogen synthesis-related genes and decrease glucose levels [101]. Mice with liver-specific knockdown of Hepatocyte Nuclear Factor 4α (HNF4α) exhibited impaired glycogen synthesis and MAFLD progression [102]. Glycogen synthase kinase 3β (GSK3β) is one of the key regulatory molecules of glycogen metabolism, which can block glycogen synthesis through the inhibition of glycogen synthase when activated by dephosphorylation, leading to hepatic insulin resistance [103]. Therefore, GSK3β is a potential candidate target for the treatment of MAFLD. Glycogen metabolism is closely related to insulin signaling [104]. Improving impaired glycogen metabolism in hepatocytes may help alleviate insulin resistance and provide new therapeutic options for alleviating MAFLD.

##### Glycolysis and MAFLD

Glycolysis is a metabolic process in which glucose is catalyzed by enzymes to produce pyruvate and other intermediates [105]. Enhanced hepatic glycolysis is an important metabolic shift in MAFLD progression [106]. Hexokinase 2 (HK2), phosphofructokinase 1 (PFK1), and pyruvate kinase type M2 (PKM2) play crucial roles in regulating glycolysis (Figure 1). Glycolysis is critical for the physiological activity of hepatic macrophages. A high-fat diet can lead to MAFLD by activating Caspase 11, which in turn enhances glycolysis and induces pyroptosis in hepatic macrophages [107]. PKM2, the key enzyme in glycolysis, can induce hepatic steatosis by driving hepatic metabolic reprogramming and hepatic macrophage M1 activation [108]. Another study showed that G protein-coupled receptor (GPCR) activation mediated PKM2 upregulation, which in turn enhanced glycolysis in Kupffer cells and suppressed hepatic inflammation, thereby alleviating high-fat diet-induced obesity and MAFLD [109]. The activation of hepatic stellate cells (HSCs) is considered to be a key mechanism leading to liver fibrosis in MAFLD, and enhanced glycolysis contributes to HSCs activation and promotes the expression of fibrosis-related genes [110,111,112,113]. More importantly, over-enhanced glycolysis in hepatocytes predicts the tendency toward hepatocellular carcinoma, as tumor cells prefer to break down glucose into lactate via glycolysis to meet the energy demands for rapid proliferation, namely, the Warburg effect [114]. In conclusion, the pathophysiology of glycolysis in MAFLD progression is complex because of the abundance of cell types in the liver. Targeting glycolysis in the liver may be a potential therapeutic strategy for MAFLD and is important for blocking MAFLD from being more malignant.

##### Gluconeogenesis and MAFLD

In contrast to glycolysis, gluconeogenesis is an enzyme-catalyzed metabolic process that converts non-carbohydrate substrates, such as lactate, amino acids, and glycerol, to glucose, which is regulated by a variety of hormones, transcription factors, and so on [115]. Glucose-6-phosphatase (G6Pase), fructose-1,6-bisphosphatase (Fbpase), pyruvate carboxylase (PC), and phosphoenolpyruvate carboxykinase (PEPCK) are the key metabolic enzymes involved in gluconeogenesis (Figure 1). Mechanistically, gluconeogenesis is an important regulatory pathway for remodeling hepatic lipid homeostasis. Glucagon is an important hormone that promotes hepatic gluconeogenesis. In patients with MAFLD, the effect of glucagon on the liver may be partially impaired, leading to “glucagon resistance” [116]. Short-term and periodic therapy with glucagon is a promising strategy for alleviating MAFLD [117]. Mice with inhibition of intestinal gluconeogenesis exhibited increased hepatic lipid uptake and de novo lipogenesis, thereby increasing the risk of MAFLD [118]. Notably, over-enhanced gluconeogenesis further contributes to hepatic insulin resistance and promotes de novo lipogenesis, which is involved in MAFLD progression from steatosis to NASH and fibrosis, and ultimately increases the risk of cirrhosis and hepatocellular carcinoma [119]. Recent studies have revealed the important role and mechanism of inhibiting hepatic gluconeogenesis in ameliorating steatosis and alleviating MAFLD [120,121]. Therefore, the role of gluconeogenesis in the pathogenesis and treatment of MAFLD is inconclusive, and further studies are needed to determine the significance of gluconeogenesis in MAFLD progression.

#### 2.1.3. Amino Acid Metabolism and MAFLD

Amino acids, as some of the essential macronutrients, play a vital role in maintaining normal physiological functions and metabolism. The liver is a key organ for protein and amino acid biosynthesis and catabolism. Considerable evidence suggests that amino acid metabolism disorders are another important pathogenesis leading to MAFLD.

##### Branched-Chain Amino Acid Metabolism and MAFLD

Branched-chain amino acids (BCAAs) contain three types of amino acids including valine, leucine, and isoleucine, which are responsible for energy supply during fasting and protein synthesis as substrates or signaling molecules [122,123]. BCAAs cannot be synthesized endogenously in animals and can only be ingested through the diet. BCAA levels in the bloodstream reflect the balance between amino acid intake and amino acid storage (i.e., proteins), and play an important role in regulating food intake and protein metabolism [123]. BCAAs enter the tricarboxylic acid (TCA) cycle to produce ATP after transamination and decarboxylation, in which branched-chain aminotransferases (BCATs) and branched-chain α-ketoacid dehydrogenases (BCKDs) are necessary [124]. Disturbed BCAA metabolism is one of the important pathological features of MAFLD. Plasma BCAA concentrations are elevated in patients with MAFLD [125,126]. Notably, there exists a gender dependence between plasma BCAA concentration and the severity of MAFLD [127]. Plasma BCAA levels were positively correlated with the grade of hepatic steatosis in children and adolescents with MAFLD [128]. This may be related to the impaired catabolism of BCAAs. In addition, elevated plasma levels of branched-chain α-keto acids (BCKAs), an intermediate metabolite of BCAAs, were strongly associated with more severe MAFLD [129]. This suggests that BCAA catabolism may be enhanced in advanced MAFLD. A recent study has shown that the enhanced valine/3-hydroxyisobutyric acid metabolic pathway mediated by 3-hydroxyisobutyryl coenzyme A hydrolase (HIBCH) is associated with MAFLD pathogenesis [130]. BCAA supplementation may alleviate hepatic steatosis and liver injury induced by a choline-deficient high-fat diet in mice by inhibiting the FAS gene and protein expression [131]. However, there is also evidence that BCAA supplementation promotes apoptosis and increases hepatocyte susceptibility to lipotoxicity in high-fat diet-fed mice [132]. In conclusion, the pathophysiological roles of BCAAs in different MAFLD stages of may differ, and the safety of preventing and controlling MAFLD through BCAA supplementation needs to be further evaluated.

##### Aromatic Amino Acid Metabolism and MAFLD

Aromatic amino acids (AAAs) contain benzene rings in their side-chain and consist of three types of amino acids including tyrosine, tryptophan, and phenylalanine. Dietary intake of AAAs is positively correlated with hepatic lipid content and iron concentration [133]. Serum tryptophan levels are significantly elevated in patients with steatohepatitis, and the levels of tryptophan and tyrosine are positively correlated with the levels of serum total cholesterol and low-density lipoprotein cholesterol [134]. Tyrosine is a necessary raw material for the synthesis of thyroid hormones such as triiodothyronine (T3) and tetraiodothyronine (T4) that regulate metabolism and growth [135]. Excessive tyrosine intake may lead to increased synthesis of thyroid hormones, thereby affecting systematic metabolism. Thyroid hormone receptor β subtype (THRβ) is the main form expressed in the liver and promotes hepatic lipid uptake and synthesis (Figure 1) [136]. Free T3 levels have been found to be positively correlated with the severity of MAFLD [137,138]. Tyrosine deficiency can lead to hepatic steatosis by interfering with hepatocellular VLDL assembly and thereby contributing to hepatic steatosis [139]. In addition, the phosphorylation of the protein tyrosine site is significant for MAFLD progression. Therefore, molecules that regulate tyrosine phosphorylation of target proteins can be potential therapeutic targets for MAFLD, such as receptor tyrosine kinases (RTKs) including epidermal cell growth factor receptor (EGFR), fibroblast growth factor receptor (FGFR), and others [140]. AAA supplementation reduced serum and hepatic TG and serum LDL-cholesterol levels and improved hepatic steatosis in mice [141]. Tryptophan can be metabolized by intestinal flora to produce indolepropionic acid (IPA), which ameliorates hepatic inflammation and injury in steatohepatitis rats by inhibiting endotoxin production [142]. Furthermore, tryptophan can be transformed into nicotinamide adenine dinucleotide (NAD), which is an active form of vitamin B_3_, through the kynurenine pathway that primarily occurs in the liver, thereby posing a significant impact on hepatic vitamin metabolism [143]. Therefore, AAAs can interact with other metabolic pathways and are crucial factors in the pathogenesis of MAFLD.

### 2.2. Micronutrient Metabolism and MAFLD

Micronutrients including vitamins and minerals are involved in vital biochemical reactions. A large amount of evidence suggests that imbalances in micronutrient metabolism are highly associated with MAFLD.

#### 2.2.1. Vitamin Metabolism and MAFLD

Vitamins are categorized into fat-soluble vitamins (vitamins A, D, E, and K) and water-soluble vitamins (vitamins B, C, etc.) according to their solubility, which are essential micronutrients for the maintenance of normal physiological functions. This section mainly focuses on the correlation between vitamins A, B, C, D, and E and MAFLD.

##### Vitamin A Metabolism and MAFLD

Vitamin A (Retinoid) refers to all-trans-retinol (or structural analogs) and metabolites such as retinyl esters, retinol, and retinoic acid, which is essential for humans [144,145]. Retinoic acid receptor (RAR), retinoid X receptor (RXR), and PPAR mediate the physiological activity of vitamin A, of which all-trans-retinoic acid (at RA) is the main active form of vitamin A (Figure 1) [145,146]. The liver (especially the HSCs) is the major place for vitamin A storage and metabolism. The activation of HSCs is accompanied by the complete loss of lipid droplets containing vitamin A and increased extracellular matrix production [144,145]. Vitamin A is stored in HSCs as retinyl esters. Mechanistically, disturbances in vitamin A metabolism lead to the excessive accumulation of retinyl esters in hepatocytes and a decrease in total retinol levels in the liver, which results in hepatic steatosis [147]. Epidemiologic evidence has shown that inadequate vitamin A intake is a significant risk factor for MAFLD and that serum retinol levels are significantly lower in patients with advanced MAFLD [148,149,150]. However, there is also evidence that MAFLD progression may be associated with elevated serum vitamin A levels [151,152]. In addition, vitamin A metabolites, especially retinoic acid, influence the development of MAFLD mainly by affecting the regulatory network of hepatic glucose–lipid metabolism, as well as the interaction with insulin [153,154]. Signaling pathways regulated by RAR and RXR are important molecular mechanisms for vitamin A to work. RARβ agonists can inhibit hepatic steatosis and fibrosis in MAFLD mice [155]. RARα in adipocytes can prevent MAFLD by inhibiting adipogenesis and inflammation and inducing energy expenditure [156]. Overall, correcting metabolism disorders of vitamin A is a promising therapeutic strategy for MAFLD.

##### Vitamin B Metabolism and MAFLD

Vitamin B contains eight types of compounds that are key cofactors in a variety of enzymatic reactions and are involved in catabolism and anabolism (Figure 1) [157]. Vitamin B_1_ (Thiamine) is an essential cofactor for α-keto acid decarboxylase and transketolase, which has beneficial effects in regulating hepatic glucose and lipid metabolism, mitochondrial function, and oxidative stress [158]. Vitamin B_2_ (flavin) exists in two active forms including flavin mononucleotide (FMN) and flavin adenine dinucleotide (FAD), which interact with flavoproteins to mediate redox reactions. Decreased FAD due to vitamin B_2_ deficiency induces fatty liver disease, which is associated with the inhibition of the PPARα pathway [159]. Vitamin B_3_ contains nicotinic acid and nicotinamide, which are nutritional precursors of bioactive molecules including NAD and nicotinamide adenine dinucleotide phosphate (NADP) [160]. Recent epidemiologic evidence shows that high dietary niacin intake can reduce hepatic steatosis and lower all-cause mortality in patients with MAFLD [161,162]. Mechanistically, niacin may ameliorate steatosis and steatohepatitis through the inhibition of DGAT2, NADPH oxidase, and NLRP3 or the activation of GPCR [163,164,165]. Nicotinamide can attenuate diet-induced steatohepatitis, oxidative stress, and liver injury in rats by decreasing the NADPH/NADP ratio and elevating glutathione levels [166,167]. Vitamin B_6_ includes pyridoxine, pyridoxal, and pyridoxamine. Pyridoxamine can inhibit the activation of HSCs and hepatic lipid peroxidation to ameliorate advanced MAFLD [168]. However, epidemiologic evidence also suggests a positive correlation between vitamin B6 intake levels and the degree of hepatic steatosis in patients with MAFLD [169]. Recent studies revealed that elevated circulating homocysteine levels and reduced vitamin B9 levels are risk factors for MAFLD [170,171]. Vitamin B_9_, also known as folate, is mainly involved in one-carbon metabolism in biochemical reactions such as nucleic acid synthesis, methylation reactions, and sulfur-containing amino acid metabolism, and insufficient intake or metabolic disorders of folate may promote MAFLD progression [172,173]. Folate and vitamin B_12_, also known as cobalamin, are key cofactors in one-carbon metabolic reactions including homocysteine metabolism. Folate was superior to other donors in ameliorating disorders of hepatic one-carbon metabolism in high-fat-fed MAFLD mice and exhibited anti-steatosis and insulin-sensitizing effects [174,175,176,177]. In addition, folate shifted to mitochondrial and consumed α-linolenic acid to maintain TGFβ1 signaling, thereby promoting HSCs activation-mediated hepatic fibrosis in MAFLD [178]. Low vitamin B_12_ levels promote lipogenesis and inhibit mitochondrial β oxidation in HepG2 cells, leading to lipid accumulation [179]. Dietary supplementation with folate and vitamin B_12_ alleviates steatohepatitis by promoting the enzymatic conversion of homocysteine [180]. A randomized controlled trial also showed the beneficial effects of vitamin B_12_ supplementation in patients with MAFLD [181]. However, reports about the relationship between vitamins B_5_ and B_7_ and MAFLD are very limited. Collectively, the vitamin B family interacts with other nutrient metabolic pathways because of their crucial roles in energy metabolism and redox reactions. However, the pathophysiology of vitamin B remains complex and unclear, and further studies are needed to explore their effects on MAFLD.

##### Vitamin C and Vitamin E Metabolism and MAFLD

Vitamin C and vitamin E, also known as ascorbic acid and tocopherol, respectively, play significant roles in the redox system because of their excellent antioxidant activity. Abundant epidemiologic evidence has demonstrated that a high vitamin C intake level is a protective factor for MAFLD [182,183,184]. Vitamin C deficiency increases hepatic steatosis, oxidative stress, fibrosis, and inflammation, which is an important potential pathogenic factor of MAFLD [185]. Vitamin C intervention significantly attenuated diet-induced MAFLD in mice, which was partly achieved by PPARα activation and the upregulated expression of its target gene involved in β-oxidation [186,187,188]. Vitamin C may also exert anti-MAFLD effects by attenuating TNFα-induced stress in hepatocytes through the activation of the FGF21/FGFR2/adiponectin pathway [189]. However, there is also evidence that chronic vitamin C deficiency impairs SREBP1c activation, thereby inhibiting hepatic de novo lipogenesis and suppressing MAFLD progression [190]. Notably, vitamin C also plays an important role in maintaining iron homeostasis and is strongly associated with MAFLD [184]. This reflects the crosstalk between vitamins and minerals. Inadequate vitamin E intake is also a risk factor for MAFLD [191,192]. Oxidative stress, namely, the imbalance between ROS production and antioxidant defense, is highly correlated with MAFLD pathogenesis and is also the main target pathway of vitamin E [193]. In addition to its antioxidant effects, vitamin E exerts beneficial effects on MAFLD through non-antioxidant actions such as the induction of adiponectin, inhibition of inflammatory signals, and modulation of macrophage M1/M2 polarization [193,194]. Taken together, the antioxidant properties of vitamins C and E have been widely noted, and their other physiological functions are worth exploring, which will contribute to understanding the role of micronutrients in MAFLD (Figure 1).

##### Vitamin D Metabolism and MAFLD

The physiological functions of vitamin D, also known as calcitriol, are not simply limited to the well-known effects on calcium homeostasis regulation. The 1,25-dihydroxy vitamin D/vitamin D receptor (VDR) axis can influence MAFLD progression by modulating a variety of pathways such as hepatic insulin resistance, oxidative stress, inflammation, and fibrosis, as well as regulating various hepatic cells such as hepatocytes, HSCs, and macrophages (Figure 1) [195,196]. In addition, vitamin D regulates the expression of several microRNAs, which are closely related to MAFLD pathogenesis [197]. Epidemiologic evidence has shown that an elevated level of serum 25-hydroxy vitamin D, the major circulating form of vitamin D, is negatively related to MAFLD and all-cause mortality [198,199,200]. Mechanistically, vitamin D exerts a therapeutic effect on MAFLD by improving hepatic lipid metabolism by regulating the PPARα and SREBP1c signaling pathways [199,201]. Recent studies have shown that VDR improves aging-related MAFLD by positively regulating mitochondrial function [202]. Additionally, the improvement of gut microbes is considered to be another potential mechanism by which vitamin D alleviates high-fat diet-induced MAFLD [203]. Overall, the mechanism by which vitamin D works is inextricably linked to other metabolic pathways, and understanding the physiopathology of vitamin D will help unravel the mystery of MAFLD.

#### 2.2.2. Mineral Metabolism and MAFLD

In addition to vitamins, minerals such as iron, copper, zinc, and selenium are also essential micronutrients for humans. Similar to most vitamins, minerals serve as cofactors for enzymes, especially those in the redox system, to carry out their physiological functions. Specifically, iron and copper have unique regulatory effects. Disturbed mineral metabolism in the liver is another important pathogenesis of MAFLD.

##### Iron Metabolism and MAFLD

Iron is a crucial metallic element that is involved in oxygen transport, DNA synthesis, ATP production, and other important biological processes [204]. The liver functions as the major organ for iron metabolism, and disturbed iron homeostasis is closely related to MAFLD. Elevated serum ferritin, iron, transferrin saturation, and hepatic iron overload are important risk factors for MAFLD [205,206]. A high-fructose diet can induce hepatic iron deposition in MAFLD accompanied by the upregulation of iron storage-related proteins and downregulation of iron export proteins [207]. Increased hepatic iron levels can lead to oxidative stress and exacerbate hepatic insulin resistance [208]. Iron overload may also lead to chronic inflammation in the liver via the activation of the cGAS-STING pathway [209]. In addition, iron accumulation can result in the over-production of ROS, which activates HSCs and induces a fibrotic phenotype [210]. More importantly, elevated hepatic iron and oxidative stress even precede typical events in MAFLD such as lipid accumulation and insulin resistance [211]. It is evident that hepatic iron homeostasis imbalance is present throughout almost the entire progression of MAFLD.

Notably, Dixon et al. reported an iron-dependent and non-apoptosis-regulated cell death due to lipid peroxide accumulation in 2012 and named it ferroptosis [212]. As mentioned before, the liver functions as a central organ for lipid metabolism. Based on the unique physiological characteristics of the liver, ferroptosis has received much attention in recent years as an MAFLD pathogenesis (Figure 1) [213]. Numerous studies have shown that ferroptosis is involved in different MAFLD stages [214]. More importantly, ferroptosis may function as a potential event triggering steatohepatitis [215]. Polyunsaturated fatty acids (PUFAs) are the most significant substrates for lipid peroxidation during ferroptosis. PUFAs are transformed into phospholipid hydroperoxides (PUFA-PL-OOHs) catalyzed by enzymes such as ACSL4, which is accelerated by ferrous ions (Fe^2+^) through the Fenton reaction and ultimately leads to the disruption of membrane structure [216]. However, monounsaturated fatty acids (MUFAs) are less oxidizable than PUFAs and inhibit lipid peroxidation and ferroptosis [217]. As mentioned above, MUFAs can be synthesized via DNL, and inhibiting the hepatic DNL and suppressing ferroptosis appear to be innately contradictory as therapeutic strategies for MAFLD. However, ferroptosis involves various regulatory mechanisms such as iron homeostasis regulation, lipid metabolism, and antioxidant defense system, as well as multiple organelles such as the endoplasmic reticulum, mitochondria, and peroxisomes [218]. The main antioxidant mechanisms in ferroptosis include Xc-/GSH/GPX4 and NAD(P)H/FSP1/CoQ10, which are responsible for the scavenging of lipid peroxides [219]. Therefore, targeting different aspects of ferroptosis provides diverse options for the prevention and treatment of MAFLD.

##### Copper Metabolism and MAFLD

Copper serves as a key component of many proteins as well as cofactors of oxidoreductase enzymes, such as ceruloplasmin (Cp), superoxide dismutase 1 (SOD1), and cytochrome C oxidase [220]. The liver is also an important place for copper metabolism. Copper deficiency leads to reduced hepatic antioxidant defenses and mitochondrial dysfunction, while iron overload may be an intermediate mechanism that impairs copper load [221,222]. This suggests a metabolic association between copper and iron. Reduced hepatic copper contents were observed in patients with MAFLD and associated with more pronounced steatohepatitis [223]. Copper deficiency was also observed in the livers of mice and rats with diet-induced MAFLD and exacerbated hepatic steatosis and liver injury [224,225,226]. Mechanistically, copper deficiency inhibits AMPK and thus leads to impaired mitochondrial biogenesis and fatty acid oxidation, in turn resulting in MAFLD (Figure 1) [227]. Cp is a protein containing copper that is secreted by hepatocytes, and hepatocyte-specific ablation of Cp reduces lipid accumulation, inhibits inflammation, attenuates fibrosis, and ameliorates liver injury, which may be associated with the remodeling of bile acid metabolism [228]. Furthermore, copper overload leads to lipogenesis, oxidative stress, and mitochondrial dysfunction, which induce hepatic steatosis and lipotoxicity [229,230]. Similarly, copper overload also mediates the programmed cell death known as cuproptosis, which is characterized by Cu^+^-mediated lipoacylation and the aggregation of enzymes that regulate the mitochondrial TCA cycle, particularly dihydrolipoamide S-acetyltransferase (DLAT), and loss of Fe-S clusters [231,232]. Ferroptosis and cuproptosis overlap in pathways that regulate complex metabolic processes including mitochondrial respiration, the TCA cycle, and GSH synthesis [233]. Several studies to date have attempted to explore the potential involvement of cuproptosis in MAFLD [234,235,236]. Therefore, the maintenance of copper homeostasis is crucial for normal physiology of the liver, and regulating copper metabolism appears to be a novel therapeutic strategy for MAFLD.

##### Zinc Metabolism and MAFLD

Zinc also functions as a cofactor of many enzymes and components of proteins involved in antioxidant, anti-inflammatory, and apoptotic activities [237]. Epidemiologic evidence has shown that reduced serum zinc levels are negatively correlated with the degree of hepatic steatosis, insulin resistance, and fibrosis [238,239,240]. One of the important mechanisms by which zinc deficiency leads to disturbed protein metabolism in patients with chronic liver disease is diminished ammonia metabolism, which may potentially induce or exacerbate endoplasmic reticulum stress and apoptosis, thereby triggering MAFLD [237,241]. Zinc supplementation attenuates high-fat diet-induced hepatic steatosis and liver injury, which is achieved partly by improving glucose–lipid metabolism disorders, but zinc intervention cannot alleviate MAFLD caused by a high-fat diet [242,243]. Nonetheless, a randomized clinical trial observed elevated zinc levels and reduced hepatic enzyme levels in serum after zinc supplementation in patients with overweight/obesity and MAFLD [244]. Recent studies also excellently characterized the roles of zinc finger proteins (ZFPs) in MAFLD. Zinc finger proteins can act as transcription factors that regulate HSCs activation, mitochondrial autophagy, the inflammatory response, and glucose–lipid metabolism, which influence MAFLD progression (Figure 1) [245,246,247,248,249]. In addition, zinc is a key component of matrix metalloproteinases (MMPs) that degrade the extracellular matrix, of which upregulated MMP14 and inactivated MMP11 are associated with hepatic steatosis, while upregulated MMP2 and MMP9 are associated with hepatic fibrosis and inflammation, respectively [250]. The coexistence of hepatic zinc deficiency and iron accumulation contributes to the development of hepatic fibrosis, which may be related to extracellular matrix remodeling and changes in lipid composition [251]. Notably, zinc was shown to be involved in ferroptosis, although its role is controversial [252,253]. Overall, this suggests a crosstalk between zinc and iron metabolism. However, the exact mechanism remains unclear. Targeting zinc metabolism is one of the promising therapeutic strategies for MAFLD.

##### Selenium Metabolism and MAFLD

Selenium is another essential trace element. Selenoproteins mediate the physiological functions of selenium, such as glutathione peroxidases (GPXs) and thioredoxin reductases (TXNRDs), which are responsible for antioxidation, anti-inflammation, and regulating immunity [254,255]. Serum levels of selenoprotein P, a selenium transporter from the liver to other tissues, were significantly elevated in patients with MAFLD and positively correlated with the severity of the disease [256]. Hepatic selenium deficiency causes redox imbalance and an inflammatory response leading to MAFLD [257]. Dietary selenium supplementation is effective against high-fat diet-induced liver injury, insulin resistance, and oxidative stress [258]. This is associated with an improvement in lipid metabolism and an enhancement in endogenous antioxidant mechanisms [259]. A bioinformatics analysis showed that reduced selenoprotein levels in MAFLD livers are accompanied by disturbed gene expression related to iron metabolism [260]. This suggests a correlation between hepatic selenium and iron metabolism. Actually, GPX4, the crucial protein in ferroptosis for scavenging lipid peroxides, is significantly regulated by selenium availability (Figure 1) [261]. Thus, the therapeutic effect of selenium supplementation on MAFLD may be partly achieved by inhibiting ferroptosis. However, epidemiologic evidence about the association between blood selenium levels and MAFLD appears to be contradictory [262,263,264]. Given the unique features of selenium, it is an attractive avenue to explore the role of selenium in MAFLD pathogenesis.

## 3. Natural Products against MAFLD via the Modulation of Nutrient Metabolism

Despite the absence of available drugs for clinical use to date, the endeavors in searching for therapeutic strategies for MAFLD continues unabated. In recent years, the various biological activities of natural products and their potential in disease treatment have attracted much attention from researchers and represent a novel strategy for drug development. Indeed, many studies have reported the beneficial effects of natural products in alleviating MAFLD, especially in ameliorating the disorders of nutrients metabolism. Therefore, we summarize the natural products against MAFLD that improve the metabolic disorders of the aforementioned nutrients in Table 1.

### 3.1. Natural Products and Lipid Metabolism in MAFLD

#### 3.1.1. Natural Products and Lipid Uptake in MAFLD

Many natural active ingredients have demonstrated potential in modulating hepatic lipid uptake. Z-ligustilide and n-Butylidenephthalide in the aqueous extract from *Angelica tenuissima* inhibited high-fat diet-induced hepatic steatosis in mice and oleic acid-induced lipid accumulation in HepG2 cells, which were correlated with downregulated CD36 and FATP5 and thus reduced lipid uptake [265]. Puerarin, a natural compound extracted from the root of *Pueraria lobata*, possesses antioxidant, anti-inflammatory, and anti-insulin resistance effects. Puerarin significantly reduced the fatty acid uptake rate in the liver by inhibiting FATP5 and CD36, which in turn ameliorated hepatic lipid accumulation, oxidative stress, and inflammatory responses in MAFLD rats [266]. The aqueous extract from the root of *Curcuma longa* L. and the ethanol extract from the root of *Liriope platyphylla* also alleviated hepatic steatosis in high-fat diet-induced obese mice through similar mechanisms [328,329]. The polyphenolic compounds including ferulic acid and p-coumaric acid from *Setaria italica* alleviated MAFLD mainly by downregulating FABP and CD36 [267]. Recent studies have also reported that diosgenin extracted from *Trigonella foenum-graecum* attenuates MAFLD by regulating fatty acid uptake through the inhibition of fatty acid transporters such as CD36, FATP2, and FABP, which is at least partly achieved by the SIRT6 and FXR pathways [268,269].

#### 3.1.2. Natural Products and DNL in MAFLD

Natural products also play important roles in regulating enhanced DNL in MAFLD. Naringin, present in citrus, can alleviate fructose-induced hepatic steatosis by downregulating the expression of ChREBP and SREBP-1c [270]. Total alkaloids extracted from *Corydalis saxicola Bunting* can reduce DNL-induced lipid accumulation in hepatocytes by hindering SREBP1 maturation through the activation of AMPK [330]. Diosgenin, in addition to reducing fatty acid uptake, can also inhibit hepatic DNL by activating AMPK and downregulating SREBP1c, thereby alleviating MAFLD [269,271]. Gallic acid, an abundant polyphenolic compound found in vegetables and fruits, can ameliorate fructose-induced hepatic steatosis by inhibiting hepatic DNL through AMPK-dependent mechanisms [272]. A natural flavonoid, Baicalein, also alleviated MAFLD through a similar mechanism [273]. Quercetin, another natural flavonoid, can inhibit hepatic DNL by phosphorylating and thus inactivating ACC to inhibit hepatic DNL [274]. Caffeine, a representative constituent of coffee, alleviated steatohepatitis by inhibiting AKT as well as SREBP1c-driven hepatic DNL [275]. The attractive health-promoting effects of *Capparis spinosa* such as antioxidant, anticancer, and antimicrobial activities have been attributed to the biologically active constituents it contains, such as alkaloids and polyphenols [331]. The extract from *Capparis spinosa* inhibited DNL by downregulating high-fat diet-induced SREBP1c as well as ACC, thereby ameliorating hepatic lipid accumulation and steatohepatitis [332]. In addition, it has been reported that chlorogenic acid, geniposide, and polydatin may reduce hepatic DNL by inhibiting unfolded protein response pathways instead of in an SREBP1- and ChREBP-independent manner [276].

#### 3.1.3. Natural Products and Fatty Acid Oxidation in MAFLD

The effects of natural products on ameliorating impaired FAO in MAFLD is also attractive. Schisandrin B extracted from the fruits of *Schisandra chinensis* (Turcz.) Baill can ameliorate hepatic steatosis by activating autophagy and promoting FAO through the AMPK signaling pathway [277]. Isosilybin isolated from *Silybum marianum* also alleviated MAFLD via a similar mechanism [278]. Icariin, one of the bioactive components isolated from the medicinal plant *Epimedium brevicornu Maxim*, can ameliorate hepatic steatosis in female rats with polycystic ovary syndrome by upregulating the PPARα-mediated expression of FAO-related genes such as CPT1α [279]. Whole grain foods are rich in ferulic acid, which can increase energy expenditure by upregulating the rate-limiting enzymes related to FAO and ketone biosynthesis, alleviating high-fat diet-induced MAFLD [280]. Sulforaphane, mainly derived from cruciferous vegetables, is a known natural activator of the antioxidant factor Nrf2. Sulforaphane can enhance the PPARα-mediated expression of FAO-related genes via the FGF21/FGFR1 pathway [281]. Hesperidin, which is abundant in citrus, and Formononetin, an active constituent of *Astragalus membranaceus* and other herbs, upregulated the SIRT1-mediated deacetylation of PGC1α, which in turn promoted PPARα-mediated FAO-related gene expression and alleviated MAFLD [282,283].

#### 3.1.4. Natural Products and Lipid Export in MAFLD

Several studies have reported natural products that alleviate MAFLD by regulating lipid output. Curcumin is an important natural polyphenolic compound derived mainly from *Curcuma longa* L., *Curcuma wenyujin*, and other turmeric plants [333]. The beneficial effects of curcumin in alleviating MAFLD can be explained partially by the upregulation of the VLDL precursor ApoB_100_, which in turn promotes hepatic lipid export [284]. Hyperoside, a natural flavanol glycoside, ameliorated MAFLD by upregulating apolipoprotein C3, which in turn promoted the assembly and secretion of VLDL-TG [285]. Brown rice refers to rice from which the bran and germ have not been removed. It was found that brown rice can ameliorate obesity-associated hepatic steatosis by upregulating ApoB and MTTP, which is positively associated with VLDL secretion [334]. In addition, quercetin as well as taurine restored impaired hepatic VLDL secretion and thereby ameliorated lipid accumulation, which is associated with alleviating endoplasmic reticulum stress [286,287]. In addition, the natural flavonoid chrysin also ameliorated hepatic lipid accumulation in MAFLD mice by increasing VLDL secretion in an HNF4α-dependent manner [288].

### 3.2. Natural Products and Carbohydrate Metabolism in MAFLD

#### 3.2.1. Natural Products and Carbohydrate Uptake in MAFLD

Many natural products may improve insulin resistance by restoring impaired glucose uptake. Berberine, an isoquinoline alkaloid compound extracted from traditional Chinese herbs, such as *Coptis chinensis*, can improve the glucose uptake rate of HepG2 cells cultivated in high-glucose condition [335]. Berberrubine, a major metabolite of berberine in the liver, promoted glucose uptake and ameliorated high-fat-induced MAFLD by increasing the expression level of GLUT2 [289]. Astragaloside IV, the main active ingredient in *Astragalus membranaceus* (Fisch.) Bunge, can effectively enhance glucose uptake in insulin-resistant HepG2 cells induced by oleic acid [290]. Triterpenoid acids-rich fractions from *Cyclocarya paliurus* (Batalin) Iljinsk. significantly increased tyrosine phosphorylation levels of insulin receptor substrates as well as 2-deoxyglucose uptake in HepG2 cells and primary hepatocytes cultivated in high-fat conditions [336]. Protocatechuic Acid and Epicatechin, which are the main constituents in the aqueous extracts from cocoa shells, restored downregulated GLUT2 levels in HepG2 cells and thus improved glucose tolerance through FGF21 signaling transduction [291]. Coniferaldehyde can also reverse impaired glucose uptake caused by high-fat diets by upregulating GLUT2 expression [292]. Skatole, a natural compound produced by plants and intestinal microbes, improved glucose uptake and insulin resistance in HepG2 cells induced by lipotoxicity [293].

#### 3.2.2. Natural Products and Glycogen Metabolism in MAFLD

Hepatic lipid accumulation is usually accompanied by a decrease in glycogen storage, and some natural products may improve MAFLD by reversing this change. Acacetin, a natural flavonoid, significantly increased glycogen distribution and content in the livers of high-fat diet-induced obese mice and improved MAFLD [294]. Aqueous extracts from *Scolymus hispanicus* L. (golden thistle) as well as Bee bread from *Heterotrigona itama* can also elevate hepatic glycogen reserves reduced by a high-fat diet [337,338]. Bavachin, a flavonoid isolated from the seeds and fruits of *Psoralea corylifolia* L., significantly increased the phosphorylation level of GSK3β in mouse primary hepatocytes and Huh 7 cells, which led to an increase in glycogen synthesis and improved insulin resistance [295]. The combined treatment of genistein, a natural phytoestrogen, or chlorogenic acid, a phenolic compound commonly found in many foods and herbs, with metformin, respectively, ameliorated MAFLD, which was partially achieved by upregulating GSK-3β phosphorylation levels and thereby enhancing glycogen synthesis [296,297]. In addition, triterpenoid acids-rich fractions from *Cyclocarya paliurus* (Batalin) Iljinsk., coniferaldehyde, and icaritin can also restore impaired glycogen synthesis and ameliorate MAFLD by upregulating the phosphorylation level of GSK3β [292,298,336]. The natural compounds glycyrrhizic acid and calycosin, on the other hand, can promote glycogen synthesis by upregulating GSK3β protein levels [299,300].

#### 3.2.3. Natural Products and Glycolysis in MAFLD

Many studies reported natural products that improve MAFLD by modulating glycolysis in the liver. Tetrahydropalmatine, an important active constituent of *Corydalis yanhusuo*, can improve hepatic steatosis by restoring the balance between glycolysis and mitochondrial oxidation, which is achieved by activating AMPK [301]. Lapachol, a naphthoquinone derived from teff, ameliorated the inflammatory response in MAFLD by inhibiting the phosphorylation of PKM2, a key enzyme in glycolysis, which in turn inhibited hepatic macrophage M1 activation [302]. Indole, a metabolite of intestinal microbes, can exert anti-hepatic steatosis and inflammation effects through a similar mechanism, except that that indole inhibits glycolysis via the activation of fructose-2,6-bisphosphatase 3 (PFKFB3) [339]. Rotundic acid, a natural triterpenoid and the main active ingredient in the bark of *Ilex rotunda Thunb.*, can inhibit the production of lactic acid from glycolysis and thereby reduce high-fat diet-induced hepatic inflammation and excessive lipogenesis [303]. Costunolide represents a promising compound for blocking fibrosis progression in MAFLD by suppressing aerobic glycolysis through the inhibition of HK2 and thus inactivating HSCs [304]. In addition, curcumin can inhibit glycolysis-mediated HSCs activation in an AMPK activation-dependent manner, providing new insights into anti-hepatic fibrosis therapy [305].

#### 3.2.4. Natural Products and Gluconeogenesis in MAFLD

Several studies also reported that some natural products could regulate gluconeogenesis in MAFLD. Berberrubine and glycyrrhetinic acid can downregulate the expression levels of key enzymes in gluconeogenesis such as G6Pase and PEPCK [289,299]. Eriocitrin, a natural flavonoid abundant in lemons, improves insulin sensitivity by attenuating hepatic gluconeogenesis [306]. Perillartine, a natural sweetener extracted from *Perilla frutescens*, improved lipid deposition and glucose homeostasis in hepatocytes by downregulating the expression of gluconeogenesis-related genes [307]. Fisetin, a natural flavonoid that exists in fruits and vegetables, can downregulate the expression levels of G6Pase and PEPCK and repair the high-fat diet-induced imbalance between hepatic glucose release and uptake [308].

### 3.3. Natural Products and Amino Acid Metabolism in MAFLD

A number of natural products have also been reported in the regulation of amino acid metabolism disorders in MAFLD, mainly affecting the composition of amino acids metabolites. Diosgenin can ameliorate high-fat diet-induced disorders of amino acid metabolism in the liver of MAFLD mice to a certain extent, especially AAAs. Diosgenin downregulated tyrosine metabolites such as dopamine, N-acetyldopamine and norepinephrine, tryptophan metabolites such as L-kynurenine and 5-hydroxytryptophan, and lysine metabolites such as 5-amino-3-oxohexanoic acid and Saccharopine, while it upregulated tyrosine metabolites such as tyromine [309]. Inulin, a prebiotic, was shown to attenuate hepatic steatosis by affecting tryptophan metabolism in gut microbes [340]. Schisandrin B can downregulate elevated L-glutamate and glutamyltyrosine levels in MAFLD [277]. The aqueous extract from the traditional Chinese medicine *Polygala japonica Houtt*. ameliorated steatohepatitis by modulating the histidine and tryptophan metabolic pathways in the liver as well as intestinal microbes. Specifically, the levels of histidine metabolites such as histamine, l-glutamate, and urocanic acid were downregulated, while the levels of tryptophan metabolites such as L-kynurenine and L-tryptophan were downregulated, and the levels of 3-hydroxyanthranilic acid and 5-hydroxyindole-3-acetic acid were upregulated [341].

### 3.4. Natural Products and Micronutrient Metabolism in MAFLD

#### 3.4.1. Natural Products and Vitamin Metabolism in MAFLD

Limited studies reported the effects of natural products on vitamin metabolism in MAFLD. Glycyrrhizin is a major constituent in the root of *Glycyrrhiza glabra* L. [342]. Glycyrrhetinic acid is a metabolite of glycyrrhizin in vivo and can elevate retinoic acid levels in the liver of MAFLD mice, especially at RA, thereby ameliorating disturbed vitamin A metabolism and mitigating MAFLD via downstream nuclear receptors pathways of vitamin A [310]. β Cryptoxanthin, a pro-vitamin A that exists in vegetables and algae, inhibited hepatic lipid accumulation, lipid peroxidation, and macrophage M1 activation, thereby ameliorating insulin resistance and inflammatory responses in the liver [311]. In addition, the consumption of brown rice elevated retinol levels and upregulated the expression of genes related to vitamin A signaling in mouse livers [334].

*Hibiscus sabdariffa* L. is rich in flavonoids, tannins, saponins, quinones, and triterpenoids, which can inhibit elevated homocysteine levels due to vitamin B_12_ deficiency and alleviate hepatic steatosis and inflammation [343]. Polyphenols in the shells of *Pisum sativum* L. elevated serum and liver levels of vitamin B_6_, which was achieved partially by promoting the production of vitamin B_6_ by gut microbes and thus ameliorated high-fat diet-induced hepatic oxidative stress, inflammation, and fibrosis through the activation of PPARα and the inhibition of the TLR4 inflammatory pathway [344].

The ethanolic extract from the seeds of *Sesamum indicum* L. elevated hepatic levels of vitamin C as well as antioxidants such as glutathione, thereby ameliorating high-fat diet-induced hepatic oxidative stress and inflammatory responses [345]. Similarly, hepatic vitamin C and E levels in high-fat diet-induced obese mice were significantly elevated after intervention with the ethyl acetate extract from *Lavatera cretica* L., which ameliorated hepatic lipid accumulation and oxidative damage [346].

In addition, studies have reported that tomatidine, a steroidal alkaloid present in Solanaceae, acts as an agonist of the vitamin D receptor to activate AMPK signaling, thereby alleviating hepatic lipid accumulation [312,313]. The unique properties of tomatidine are expected to attenuate the negative effects caused by vitamin D deficiency in MAFLD.

#### 3.4.2. Natural Products and Mineral Metabolism in MAFLD

Several studies have reported the role of natural products in improving iron metabolism in MAFLD. Curcumol is a sesquiterpenoid extracted from *Curcuma longa* L., *Curcuma kwangsiensis*, and other turmeric plants [347]. Curcumol can alleviate hepatocyte senescence and ameliorate MAFLD by blocking free iron release through the inhibition of ferritinophagy [314]. *Exocarpium Citri Grandis*, the unripe or nearly ripe dried outer pericarp of *Citrus grandis* L., may inhibit iron overload and alleviate lipid accumulation and liver injury by modulating the iron transport and storage capacity of hepatocytes, thereby inhibiting iron overload [348]. In addition, natural compounds such as betaine, zeaxanthin, and epigallocatechin gallate have been reported to improve MAFLD by attenuating hepatic iron deposition [315,316,317]. In addition to improving iron homeostasis, many studies have reported the inhibitory effects of natural products on ferroptosis in MAFLD. Ginkgolide B, a terpene trilactone extracted from the leaves of *Ginkgo biloba* L., inhibits iron overload and lipid peroxidation in the liver, thereby attenuating ferroptosis and alleviating MAFLD [318]. Additionally, dehydroabietic acid, quercetin, atractylodin, epimedium, arbutin, and puerarin alleviate high-fat diet-induced liver injury by upregulating the expression of antioxidant-related factors in ferroptosis, especially Nrf2 and GPX4 [319,320,321,322,323,324].

Reports on natural products regulating the metabolism of other minerals are quite limited. Oleuropein, a non-toxic cyclic enol ether terpene phenol isolated from the fruits and leaves of *Olea europaea* L., is able to ameliorate MAFLD by activating AMPK-dependent autophagy and may act as a natural chelator of copper ions to modulate hepatic copper homeostasis and inhibit oxidative stress [325,326]. Selenoneine, a natural organic selenium compound mainly found in fish tissues such as tuna, significantly increased selenium levels in mouse livers and alleviated hepatic steatosis and liver injury in mice with MAFLD [327].

## 4. Conclusions

Present knowledge identifies MAFLD as a hepatic disease closely related to metabolic disorders, and homeostasis imbalances of macronutrients such as lipids, carbohydrates, and amino acids, as well as micronutrients such as vitamins and minerals, are strongly associated with MAFLD progression. However, no drugs have been approved for the clinical treatment of MAFLD to date, which greatly compromises the medical benefits for patients. Indeed, there have been numerous attempts by researchers to utilize natural resources to develop active products beneficial for MAFLD prevention and treatment, and many natural products exhibit excellent potential to ameliorate metabolic disorders in MAFLD. However, it is significant to note that the current understanding of MAFLD pathophysiology is far from adequate, and the complicated associations among various metabolic pathways greatly challenge the exploration of pathogenesis as well as the development of drugs. While natural products have shown promising pharmacological properties, it is important to note that these findings are primarily based on animal studies and in vitro experiments. Therefore, further research is needed before they can be applied in clinical use. In addition, there are significant differences among natural products in their physicochemical nature, preparation methods, and research strategies, and their active ingredients, purity, biosafety, availability, pharmacokinetics, and efficacy must be evaluated normatively, which may be the focus for future related research.

## Figures and Tables

**Table 1:** Natural products against MAFLD through different metabolic pathways.

Compound	Chemical Structure	Potential Mechanism	Potential Targets	References
Z-ligustilide	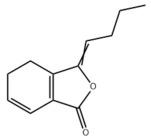	Lipid uptake	CD36, FATP5	[265]
n-Butylidenephthalide	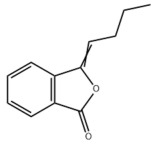	Lipid uptake	CD36, FATP5	[265]
Puerarin	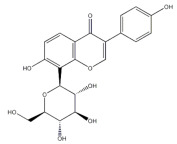	Lipid uptake	CD36, FATP5	[266]
Cerulic acid	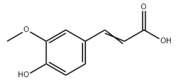	Lipid uptake	FABP, CD36	[267]
P-coumaric acid	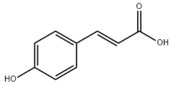	Lipid uptake	FABP, CD36	[267]
Diosgenin	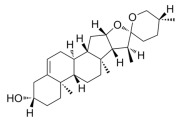	Lipid uptake	CD36, FATP2, FABP	[268,269]
Naringin	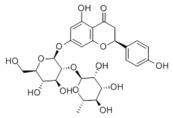	De novo lipogenesis	ChREBP, SREBP-1c	[270]
Diosgenin	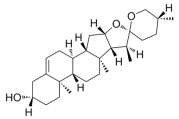	De novo lipogenesis	AMPK, SREBP-1c	[269,271]
Gallic acid	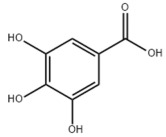	De novo lipogenesis	AMPK	[272]
Baicalein	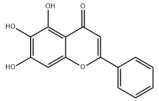	De novo lipogenesis	AMPK	[273]
Quercetin	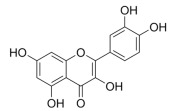	De novo lipogenesis	ACC, AMPK	[274]
Caffeine	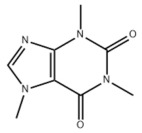	De novo lipogenesis	AKT, SREBP1c	[275]
Chlorogenic acid	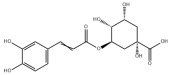	De novo lipogenesis	XBP1	[276]
Geniposide	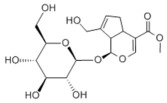	De novo lipogenesis	XBP1	[276]
Polydatin	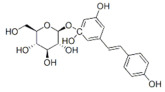	De novo lipogenesis	XBP1	[276]
Schisandrin B	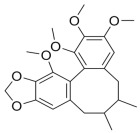	Fatty acid oxidation	AMPK	[277]
Isosilybin	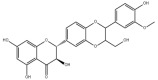	Fatty acid oxidation	AMPK, PPARα, ACOX1, CPT1α	[278]
Icariin	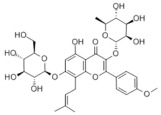	Fatty acid oxidation	PPARα, ACOX1, CYP4A3, CPT1α	[279]
Ferulicacid	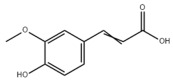	Fatty acid oxidation	CPT1α, ACOX1, HMGCS2	[280]
Sulforaphane	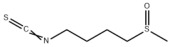	Fatty acid oxidation	FGF21, FGFR1, p38 MAPK, PPARα, CPT1α	[281]
Hesperidin	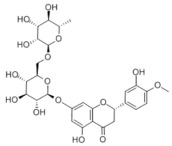	Fatty acid oxidation	SIRT1, PGC1α	[282]
Formononetin	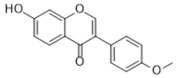	Fatty acid oxidation	SIRT1, PGC1α	[283]
Curcumin	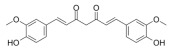	Lipid output	ApoB100	[284]
Hyperoside	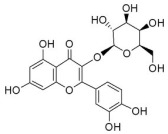	Lipid output	ApoC3, VLDL	[285]
Quercetin	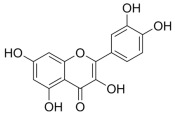	Lipid output	IRE1, XBP1s, VLDL	[286]
Taurine	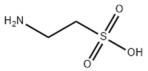	Lipid output	IRE1, XBP1s, ATF6, CHOP, MTTP, VLDL	[287]
Chrysin	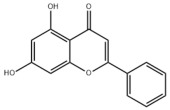	Lipid output	PKC, HNF4α, VLDL	[288]
Berberrubine	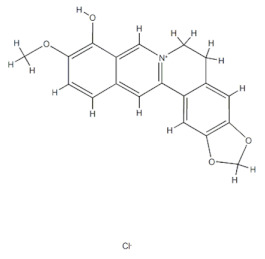	Carbohydrate intake	GLUT2	[289]
Astragaloside IV	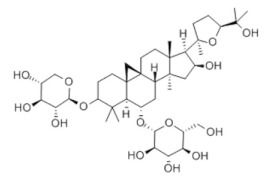	Carbohydrate intake	PTP1B, IR, IRS1	[290]
Protocatechuic Acid	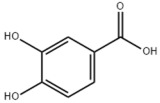	Carbohydrate intake	AKT, GLUT2	[291]
Epicatechin	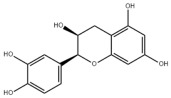	Carbohydrate intake	AKT, GLUT2	[291]
Coniferaldehyde	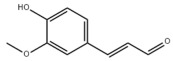	Carbohydrate intake	AMPK, GLUT2	[292]
Skatole	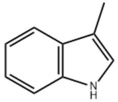	Carbohydrate intake	PERK, IRE1, etc.	[293]
Acacetin	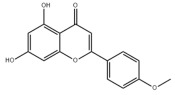	Glycogen synthesis	AMPK	[294]
Bavachin	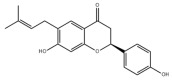	Glycogen synthesis	AKT, GSK3β	[295]
Genistein	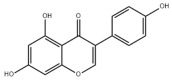	Glycogen synthesis	GSK3β	[296]
Chlorogenic acid	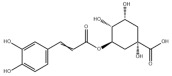	Glycogen synthesis	GSK3β	[297]
Coniferaldehyde	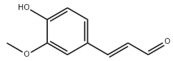	Glycogen synthesis	GK	[292]
Icaritin	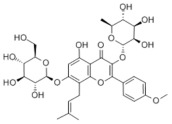	Glycogen synthesis	AKT, GSK3β	[298]
Glycyrrhizic acid	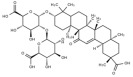	Glycogen synthesis	GSK3β	[299]
Calycosin	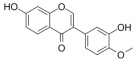	Glycogen synthesis	GSK3β	[300]
Tetrahydropalmatine	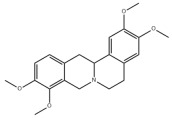	Glycolysis	AMPK	[301]
Lapachol	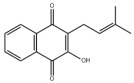	Glycolysis	PKM2	[302]
Rotundic acid	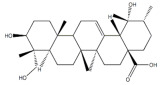	Glycolysis	TLR4, AP1	[303]
costunolide	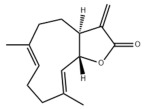	Glycolysis	HK2	[304]
Curcumin	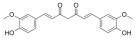	Glycolysis	AMPK, HK, PFK2	[305]
Glycyrrhizic acid	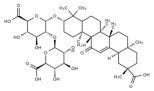	Gluconeogenesis	G6Pase, PEPCK	[299]
Berberrubine	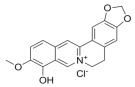	Gluconeogenesis	G6Pase, PEPCK	[289]
Eriocitrin	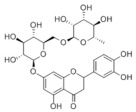	Gluconeogenesis	PEPCK	[306]
Perillartine	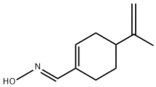	Gluconeogenesis	AKT, RORγ, G6P, PEPCK	[307]
Fisetin	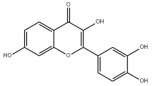	Gluconeogenesis	G6Pase, PEPCK	[308]
Diosgenin	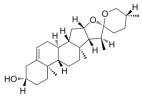	Amino acid metabolism	—	[309]
Schisandrin B	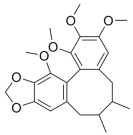	Amino acid metabolism	—	[277]
Glycyrrhetinic acid	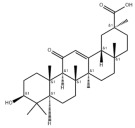	Vitamin A metabolism	AKR1B10	[310]
β cryptoxanthin	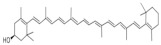	Vitamin A metabolism	IRS, etc.	[311]
Tomatidine	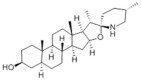	Vitamin D metabolism	VDR, AMPK	[312,313]
Curcumol	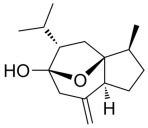	Iron metabolism	YAP, NCOA4	[314]
Betaine	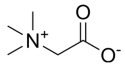	Iron metabolism	FPN, HAMP	[315]
Zeaxanthin	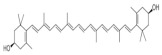	Iron metabolism	p53, GPX4, SLC7A11, SAT1, ALOX15	[316]
Epigallocatechin gallate	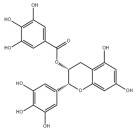	Iron metabolism	GPX4, COX2, ACSL4	[317]
Ginkgolide B	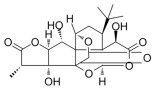	Iron metabolism	Nrf2, GPX4, HO-1, TFR1, FTH1	[318]
Dehydroabietic acid	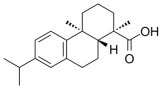	Iron metabolism	Nrf2, GPX4, HO-1, FSP1	[319]
Quercetin	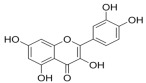	Iron metabolism	GPX4, COX2, ACSL4	[320]
Atractylodin	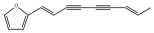	Iron metabolism	Nrf2, GPX4, FTH1, SLC7A11	[321]
Icariin	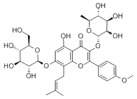	Iron metabolism	Nrf2, SLC7A11, GPX4	[322]
Arbutin	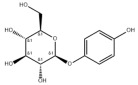	Iron metabolism	FTO, SLC7A11	[323]
Puerarin	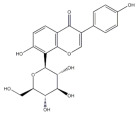	Iron metabolism	SIRT1, Nrf2	[324]
Oleuropein	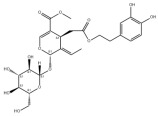	Copper metabolism	AMPK, CTR1, CTR2, ATP7B, COX17, CCS, ATOX1	[325,326]
Selenoneine	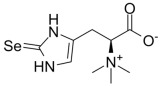	Selenium metabolism	HMOX1, GSTA1/2, GPX1, Selenoprotein	[327]

## Data Availability

No new data were created or analyzed in this study. Data sharing is not applicable to this article.

## Abbreviations

The abbreviations presented in this review are listed as follows.

Abbreviations	Full names
ACC	Acetyl-CoA Carboxylase
ACSL4	Acyl-CoA Synthetase Long-chain family member 4
ACSL5	Acyl-CoA Synthetase Long-chain family member 5
AGPAT	Acylglycerol-3-Phosphate Acyl Transferase
AhR	Arylhydrocarbon Receptor
ApoB_100_	Apolipoprotein B_100_
BCATs	branched-chain aminotransferases
BCKDs	branched-chain α-ketoacid dehydrogenases
BMP4	Bone morphogenetic protein 4
CACT	Carnitine Acyl Carnitine Translocase
CCTα	Cytidine triphosphate: phosphocholine Cytidylyltransferase-α
CD36	Cluster Determinant 36
ChREBP	Carbohydrate Response Element Binding Protein
CP	ceruloplasmin
CPT1	Carnitine Palmitoyl Transferase 1
CPT2	Carnitine Palmitoyl Transferase 2
CYP4A	cytochrome P450
DGAT	diacylglycerol acyltransferase
DLAT	dihydrolipoamide S-acetyltransferase
EGFR	epidermal cell growth factor receptor
EIF5A	Eukaryotic Initiation Factor 5A
FAO	fatty acid oxidation
FAS	Fatty Acid Synthase
FATPs	Fatty Acid Transporter Proteins
Fbpase	fructose-1,6-bisphosphatase
FGFR	fibroblast growth factor receptor
FMO2	Flavin-containing Monooxygenase 2
G6Pase	Glucose-6-phosphatase
GBE	glycogen branching enzyme
GCK	glucokinase
GLUTs	Glucose Transporter proteins
GPAT	Glycerol-3-Phosphate Acyl Transferase
GPCR	G protein-coupled receptor
GPXs	glutathione peroxidases
GSK3β	glycogen synthase kinase 3β
GYS	glycogen synthase
HIBCH	3-hydroxyisobutyryl coenzyme A hydrolase
HK2	Hexokinase 2
HNF4α	Hepatocyte Nuclear Factor 4α
INSIG2	Insulin-Induced Gene 2
LXRs	Liver X Receptors
MCJ	Methylation-Controlled J protein
MMPs	matrix metalloproteinases
mTORC1	mammalian Target of Rapamycin Complex 1
MTTP	Microsomal Triglyceride Transfer Protein
PAPs	Phosphatidic Acid Phosphatases
PAR2	Protease-Activated Receptor 2
PEPCK	phosphoenolpyruvate carboxykinase
PFK1	phosphofructokinase 1
PFKFB3	fructose-2,6-bisphosphatase 3
PKM2	pyruvate kinase type M2
PPARα	Peroxisome proliferator-activated receptor α
PPARγ	Peroxisome Proliferator-Activated Receptor γ
RAR	retinoic acid receptor
RTKs	receptor tyrosine kinases
RXR	retinoid X receptor
SCAP	SREBP Cleavage-Activating Protein
SGLT2	Sodium-dependent Glucose Transporter protein 2
SMLR1	Small Leucine-Rich protein 1
SOD1	superoxide dismutase 1
SREBPs	Sterol Regulatory Element Binding Proteins
THRβ	Thyroid hormone receptor β subtype
TM4SF5	Transmembrane 4L Six Family member 5
TM6SF2	Transmembrane 6 Superfamily member 2
TXNRDs	thioredoxin reductases
VDR	vitamin D receptor
VLDL	Very Low-Density Lipoprotein
ZFPs	zinc finger proteins
